# Association of Poor Differentiation or Positive Vertical Margin with Residual Disease in Patients with Subsequent Colectomy after Complete Macroscopic Endoscopic Resection of Early Colorectal Cancer

**DOI:** 10.1155/2017/7129626

**Published:** 2017-06-01

**Authors:** Ki Ju Kim, Hyun Seok Lee, Seong Woo Jeon, Sun Jin, Sang Won Lee

**Affiliations:** ^1^Department of Internal Medicine, Kyungpook National University School of Medicine, Daegu, Republic of Korea; ^2^Department of Internal Medicine, CHA Gumi Medical Center, CHA University, Gumi, Republic of Korea

## Abstract

In the presence of unfavorable pathologic results after endoscopic resection of colorectal cancer, colectomy is routinely performed. We determined the risk factors for residual diseases in patients with colectomy after complete macroscopic endoscopic resection of early colorectal cancer. We identified consecutive patients who underwent endoscopic resection of early colorectal cancer and subsequently underwent colectomy, from January 2011 to December 2014. Clinicopathologic risk factors related to the residual disease were analyzed. In total, 148 patients underwent endoscopic resection and subsequent colectomy. Residual disease on colectomy was noted in 16 (10.9%) patients. The rates of poorly differentiated/mucinous histology (*p* = 0.028) and of positive or unknown vertical resection margin (*p* = 0.047) were higher in patients with residual disease than in those without. In multivariate analysis, a poorly differentiated/mucinous histology and positive or unknown vertical resection margin were significantly associated with residual disease (odds ratio = 7.508 and 2.048, *p* = 0.015 and 0.049, resp.). After complete macroscopic endoscopic resection of early colorectal cancer, there is a greater need for additional colectomy in cases with a positive or unknown vertical resection margin or a poorly differentiated/mucinous histology, because of their higher risk of residual cancer and lymph node metastasis.

## 1. Introduction

The increased availability and widespread use of colonoscopy have allowed the early detection of colorectal polyps [1–3]. The proportion of polyps that contain invasive cancer is not high; nonetheless, 0.2–8.3% of them are malignant polyps, which invade through the muscularis mucosa and can metastasize to regional lymph nodes. Therefore, after endoscopic resection, colectomy may be necessary to ensure the complete removal of residual tumors in the colorectal wall and of local lymph node metastasis [4–6].

However, in clinical practice, it is challenging to identify patients who need subsequent colectomy for residual disease. Concerning the associated risk, most authors acknowledge that a positive endoscopic resection margin, poor tumor differentiation, lymphovascular invasion, and deep submucosal invasion are associated with adverse outcomes [4, 5, 7–10]. Patients with any of these high-risk factors typically undergo radical colectomy with lymph node dissection after endoscopic resection. However, most such patients have no residual disease in their surgical specimen, despite these risk factors. Specifically, the proportion of patients with residual tumor, lymph node metastasis, or recurrent tumor during follow-up is 10–13% [4].

Therefore, before a subsequent colectomy is performed in these cases, clinicians should assess the risk of residual disease against the risk of colectomy itself. In the present study, we aimed to identify the risk factors for residual cancer and lymph node metastasis in patients with subsequent colectomy after complete macroscopic endoscopic resection of early colorectal cancer.

## 2. Materials and Methods

### 2.1. Patients

**Figure 1:**
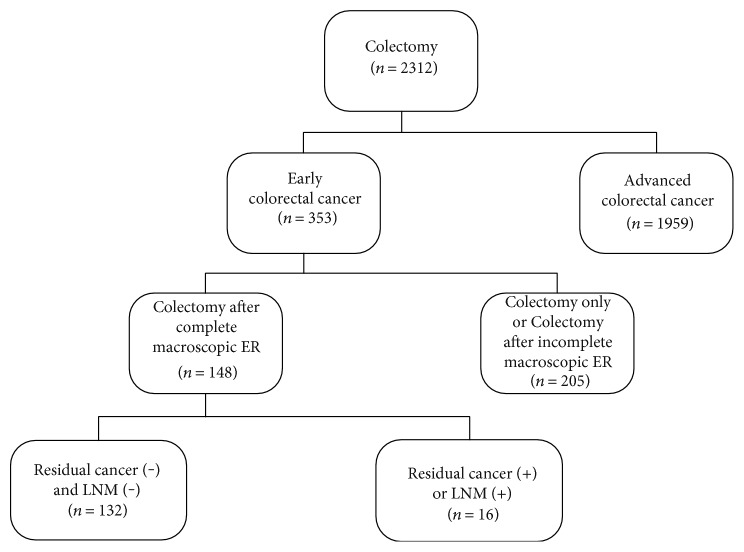
Flowchart of the patient inclusion by using the colorectal cancer database. Of 148 patients who underwent colectomy for early colorectal cancer after complete macroscopic endoscopic resection (ER), 16 showed residual cancer in the colorectal wall or lymph node metastasis (LNM) in the surgical specimen.

We examined our colorectal cancer database to find patients who had undergone colectomy for colorectal cancer at our institution between January 2011 and December 2014. In total, 2312 patients had undergone surgical treatment. Among them, 353 patients underwent colectomy for early colorectal cancer that was limited to the mucosal and submucosal layers. Ultimately, we included 148 consecutive patients who had undergone complete macroscopic endoscopic resection followed by colectomy for early colorectal cancer in this study. A flowchart of our study is shown in Figure 1. All patients had 1 or more of the following risk factors for residual disease: (i) the lesion had a poorly differentiated/mucinous histology; (ii) the vertical or lateral endoscopic resection margin was positive, or the status of the margin was unknown; (iii) lymphovascular invasion was found in the endoscopic resection specimen; or (iv) the submucosal invasion depth was >1000 *μ*m (i.e., not superficial). Endoscopic resection was considered macroscopically complete if the tumor had been removed without any visible remnant lesions on endoscopy. Patients who had undergone incomplete macroscopic endoscopic resection or those without a diagnosis of cancer, which was limited to the mucosal and submucosal layers, were excluded. The institutional review board at Kyungpook National University Medical Center approved this retrospective study.

### 2.2. Data Collection

We collected the data by reviewing the colorectal cancer database, which consisted of stored endoscopic photographs and medical records; the records included the clinical characteristics of the patients, endoscopic procedures performed, en bloc resection, tumor location, macroscopic or microscopic features, and histopathology results from surgical resection specimens.

The endoscopic resection techniques, including endoscopic mucosal resection and endoscopic submucosal dissection, were all performed at different times [11, 12]. Furthermore, the morphologic appearance of tumors was collected, as per the update to the Paris classification of superficial neoplastic lesions in the digestive tract [13].

Each endoscopic resection specimen was examined histologically to determine the tumor size, histologic type, and differentiation grade; lymphovascular invasion; status of the vertical and lateral endoscopic resection margins; and submucosal invasion depth. The endoscopic resection margin was defined as positive if the length between the deepest part of the tumor and the margin was <1000 *μ*m, or if malignant tumor cells were present within 1000 *μ*m of the margin. The submucosal invasion depth was measured by using the method proposed by Kitajima et al. [14].

Either open or laparoscopically assisted colectomy with lymph node dissection was performed in accordance with the approved standard approach. The surgical specimens were histopathologically examined to determine (i) whether any tumor remained in the colorectal wall, (ii) whether lymph node metastasis had occurred, and (iii) which postsurgical pathological stage was involved.

A postoperative follow-up assessment was performed 6 months after surgery and then annually after the initial treatment. These assessments included tests for carcinoembryonic antigen levels, abdominal computed tomography scans, and colonoscopy, and they were conducted according to the National Comprehensive Cancer Network Clinical Practice Guidelines in Oncology for colon cancer and rectal cancer, as well as their updated versions [15, 16].

### 2.3. Statistical Analysis

Data were statistically analyzed using the SPSS software package, version 18.0 (SPSS Inc., Chicago, IL, USA). Categorical variables are expressed as proportion (%), and continuous variables are expressed as median with range. Pearson's chi-square test with Fisher's exact test was used to compare the categorical variables between the residual disease group and the no residual disease group, whereas the Mann-Whitney *U* test was used to compare continuous variables. Univariate and multivariate analyses of the factors associated with residual disease were performed using a logistic regression model. For each factor, an odds ratio (OR) with 95% confidence interval (CI) was estimated. A *p* value <0.05 was considered statistically significant.

## 3. Results

### 3.1. Characteristics of Patients and Tumors

Of the 148 patients who had undergone colectomy after complete macroscopic endoscopic resection of early colorectal cancer, 144 (97.3%) had an invasion into the submucosa and the remaining 4 (2.7%) had intramucosal cancer (Table 1). The mean submucosal invasion depth was 2075.2 *μ*m (range, 300–7000 *μ*m). One hundred forty-one (95.3%) patients had well- or moderately differentiated adenocarcinoma, and the remaining 7 (4.7%) had poorly differentiated or mucinous adenocarcinoma. Lymphovascular invasion was found in 30 (20.3%) patients. Concerning the margin status of the endoscopic resection specimen, the vertical margin was positive in 48 (32.4%) patients and unknown in 20 (13.5%) patients; the lateral margin was positive in 31 (20.9%) patients and unknown in 20 (13.5%) patients.

Anterior resection was done in 69 (46.6%) patients, whereas low anterior resection was performed in 55 (37.2%) patients, right hemicolectomy in 13 (8.8%) patients, left hemicolectomy in 6 (4.1%) patients, and transverse colectomy in 5 (3.4%) patients. A residual tumor in the colorectal wall was identified in 6 (4.1%) patients, whereas regional lymph node metastasis was diagnosed in 10 (6.8%) patients. Moreover, one patient had 2 lymph node metastases and another patient had 3 lymph node metastases; therefore, 8 patients presented with only 1 lymph node metastasis. None of the patients had simultaneous residual colonic cancer and lymph node metastasis after colectomy.

Any of the 67 patients with a negative vertical and lateral resection margins had no residual tumor in the colorectal wall. Of the 48 patients with a positive vertical margin, 4 (8.3%) had residual tumor in the colorectal wall. Of the 20 patients with unknown vertical margin, 2 (10%) had residual tumor in the wall. Of the 31 patients with a positive lateral margin, 4 (12.9%) had residual tumor in the colorectal wall. Of the 20 patients whose lateral margin was unknown, 2 (10%) patients had residual tumor in the wall.

Of the 141 patients with a well- or moderately differentiated tumor, only 7 (5.0%) had node metastasis. However, of the 7 patients who had a poorly differentiated or mucinous histology, 3 (42.9%) had lymph node metastasis. Five (16.7%) of 30 patients with lymphovascular invasion on endoscopic resection had lymph node metastasis on colectomy. The sensitivity and specificity of lymphovascular invasion to predict lymph node metastasis were 50% (5 of 10) and 81.9% (113 of 138), respectively.

### 3.2. Comparison between Patients with or without Residual Disease at Colectomy

We compared the clinicopathologic characteristics between the residual disease group and the no residual disease group (Table 2). The rate of poorly differentiated/mucinous histology in the residual disease group was higher than that in the no residual disease group (18.8% versus 3.0%, *p* = 0.028). A positive or unknown vertical endoscopic margin was observed in 11 patients of the residual disease group, who had a higher rate compared with patients in the no residual disease group (68.8% versus 43.2%, *p* = 0.047).

### 3.3. Risk Factors for Residual Disease after Subsequent Colectomy

Univariate analysis showed that a poorly differentiated/mucinous histology and a positive or unknown vertical margin status were significantly associated with residual disease (*p* = 0.014 and 0.048, resp.) (Table 3). Lymphovascular invasion, submucosal invasion depth, positive or unknown lateral margin status, and other factors were not significantly associated with residual disease. In multivariate analysis, a poorly differentiated/mucinous histology (OR = 7.508, 95% CI 1.47–38.1, *p* = 0.015) and a positive or unknown vertical margin (OR = 2.048, 95% CI 1.00–4.17, *p* = 0.049) were also independent risk factors for residual disease after subsequent colectomy.

### 3.4. Follow-Up

The median follow-up period was 38 months (range, 16–63 months), and all patients undertook the postoperative follow-up program faithfully. During the follow-up period, no postoperative death occurred. Moreover, 10 patients with node metastasis underwent postoperative chemotherapy. Only 1 patient (0.7%) developed liver metastasis at 12 months after the radical colectomy with lymph node dissection and underwent liver resection with additional chemotherapy. The patient was alive at the last follow-up. In the remaining 147 patients, there was no evidence of tumor recurrence during the follow-up period.

## 4. Discussion

Endoscopic resection for early colorectal cancers has definite benefits. As it is a less invasive procedure, endoscopic resection results in reduced surgical morbidity and faster healing. The major disadvantages of endoscopic resection are the oncological outcomes associated with residual tumors in the remaining colorectal wall, and lymph node metastasis [9]. Thus, in patients with risk factors for such residual disease, subsequent colectomy with node dissection is suggested. However, it remains challenging to select patients for this radical surgery because studies attempting to determine the risk factors for residual disease have provided varying results, and they have been restricted by a small sample size and the presence of selection bias [1, 17–21].

In this study, 148 patients underwent complete macroscopic endoscopic resection of early colorectal cancer, followed by colectomy. Residual disease after colectomy was noted in 10.9% of patients: 4.1% had residual tumor in the colorectal wall and 6.8% had local lymph node metastasis. Similar to the findings in previous reports [4], about 89% of the patients had no residual tumor in the surgical specimen; that is, they underwent colectomy unnecessarily.

It is less challenging to identify patients who require colectomy to ensure the removal of residual tumor in the colorectal wall. In the current study, none of the patients with a negative resection margin had any residual tumor in the colorectal wall. About 8% of the patients with a positive vertical resection margin and about 13% of the patients with a positive lateral margin had residual tumor in the colorectal wall. Previous studies have reported that the rate of residual tumor in early colorectal cancer with negative resection margins is 0–2%. However, when the margins are positive, the rate of residual tumor is 20–34% [5, 7, 20, 22]. Notably, in this study, patients with unknown endoscopic resection margins had similar rates of residual tumor in the colorectal wall as those with positive margins (10%). This suggests that patients with unknown resection margin must be assessed in the same manner as patients with a positive margin, and an unknown margin status should be considered a risk factor for residual tumors. Therefore, it is vital that resection specimens be delivered in 1 whole slice, which means en bloc removal, so that the resection margins can be assessed properly by pathologists [6].

In addition, one study reported that histologic examinations of colectomy specimens showed no residual tumor in patients who underwent macroscopic complete endoscopic resection and showed positive lateral resection margins. Therefore, the necessity for subsequent surgery in patients with positive lateral margins remains unclear [23]. There are some reports on the feasibility and efficacy of repeat or salvage endoscopic submucosal dissection for residual or local recurrent colorectal tumors after endoscopic resection, to avoid surgical resection. One prospective study showed an R0 endoscopic resection rate of 83% without major complications and an overall curative resection rate of 96% for 30 residual or recurrent lesions [24]. A recent retrospective study also showed similar results: An en bloc resection rate of 100% and a curative resection rate of 93% in 28 patients [25]. Although these studies were limited by small patient numbers and their designs, they showed the possibility that such an approach could spare patients with involved lateral margins from undergoing a major surgery.

It is also important to identify patients who require colectomy after endoscopic resection to remove regional node metastasis; however, this is more complicated. For several decades, many studies have addressed the question of whether a patient who has undergone endoscopic resection for early colorectal cancer also requires colectomy [1, 5, 7, 8, 20]. Nonetheless, the answer remains obscure to a certain degree. One study [5] reported that poorly differentiated or mucinous adenocarcinoma occurred in 5.7–9.2% of early colorectal cancers and that the incidence of lymph node metastasis was 36–37.5%. Similar to the findings of previous reports, the current study showed that the proportion of patients with poor differentiation was 4.7% (7 of 148) and that the incidence of lymph node metastasis was 42.9% (3 of 7). Concerning the risk factors for residual disease, most investigators agree that tumor differentiation correlates with the likelihood of lymph node metastasis [4, 5, 20, 26].

In the current study, the incidence of lymphovascular invasion was 20.3%, and patients with lymphovascular invasion presented with a higher incidence of lymph node metastasis (16.7%, 5 of 30) than those without lymphovascular invasion. Therefore, although lymphovascular invasion can also function as a prognostic predictor of residual disease or recurrence, the scientific evidence is less conclusive. Some authors have shown that lymphovascular invasion is correlated with regional lymph node metastasis and recurrence [5, 8, 23, 27, 28], whereas others have reported no association [2, 7, 18, 29]. Analysis on this matter is further complicated by the fact that lymphovascular invasion is not often seen in endoscopic resection specimens of early colorectal cancer. More importantly, it is technically challenging for the pathologist to identify and interpret lymphovascular invasion because retraction artifacts often occur and the specimen sizes are small [4]. Moreover, the sensitivity and specificity of lymphatic or vascular invasion to predict lymph node metastasis are not satisfactory. In the current study, 50% of patients with nodal metastasis did not present with lymphovascular invasion and 18.1% of patients without nodal metastasis presented with lymphovascular invasion.

In this study, the most frequent reason for colectomy was deep submucosal invasion of >1000 *μ*m. Lymph node metastasis was identified in 9 of 122 of these cases (7.4%). The submucosal invasion depth did not differ significantly between patients who were positive for lymph node metastasis and those who were negative. The sensitivity of deep submucosal invasion of >1000 *μ*m to predict nodal metastasis was 90% (9 of 10). However, the risk factor had a low specificity (18.1%, 25 of 138), which meant that many patients underwent needless operation (false-positive group). One study [30] reported that 12.3% of patients with submucosal invasion of >1000 *μ*m demonstrated lymph node metastasis and that such invasion increased the risk of lymph node metastasis (risk ratio = 5.2, 95% CI 1.8–15.4). The authors added that a 1000 *μ*m cutoff point for submucosal invasion depth would ensure that lymph node metastasis-positive patients are allocated to the high-risk group with a sensitivity of 96.7%; however, the specificity is low (24.1%). Another study [31] reported that the incidence of nodal metastasis in colorectal cancer with a submucosal invasion depth of ≥1000 *μ*m was 12.5%. However, nearly 90% of patients with an invasion depth of ≥1000 *μ*m did not show nodal metastasis. Therefore, in considering whether colectomy is necessary, it is vital that clinicians take into account factors other than the submucosal invasion depth (i.e., other risk factors for residual disease, performance status, and the will of the patient).

This was a large case study on the risk factors for residual tumor in the colorectal wall, or lymph node metastasis, in patients with subsequent colectomy after complete macroscopic endoscopic resection of early colorectal cancer. However, this retrospective study had some limitations. First, only patients who had undergone colectomy after complete endoscopic removal of early colorectal cancer were included in this study, and this may have led to a selection bias. Patients who did not undergo subsequent colectomy despite the risk factors for residual disease were excluded because they were either too weak to endure surgery or did not want to undergo surgery. Nonetheless, we tried to minimize selection bias by aiming to determine the risk factors for imperfect resection in the endoscopic removal of the surgical specimen and by including all patients who had undergone surgery after endoscopic resection. Second, our study is limited by the small number of events despite the large number of cases. These limitations are common in studies that attempt to confirm assumed risk factors for residual disease on endoscopic resection of early colorectal cancers.

In conclusion, a poorly differentiated/mucinous histology and a positive or unknown vertical resection margin were risk factors for residual tumor in the colorectal wall or nodal metastasis in subsequent colectomy after complete macroscopic endoscopic resection of early colorectal cancer. Therefore, after complete macroscopic endoscopic resection of early colorectal cancer, there is a greater need for additional colectomy in cases with a positive or unknown vertical resection margin or a poorly differentiated/mucinous histology, because of their higher risk of residual cancer and lymph node metastasis than other cases. However, more studies need to be performed before these suggestions can be applied in clinical practice.

## Figures and Tables

**Table 1:** Clinicopathologic characteristics of patients who underwent colectomy after macroscopic complete endoscopic resection of early colorectal cancer (*n* = 148).

Characteristics	Value
Age, years	60 (31–78)
Men/women	102 (68.9)/46 (31.1)
Tumor location	
Cecum and ascending colon	11 (7.4)
Transverse colon	9 (6.1)
Descending colon	6 (4.1)
Sigmoid colon	71 (48.0)
Rectum	51 (34.5)
Size of tumor (histologically measured), mm	13 (4–52)
Macroscopic form of tumor	
Pedunculated	32 (21.6)
Semipedunculated	67 (45.3)
Sessile or flat	49 (33.1)
Endoscopic resection method	
EMR	141 (95.3)
ESD	7 (4.7)
En bloc resection	121 (81.8)
Histologic differentiation	
Well	57 (38.5)
Moderate	84 (56.8)
Poor	3 (2.0)
Mucinous	4 (2.7)
Depth of invasion	
Mucosa	4 (2.7)
Submucosa	144 (97.3)
Submucosal invasion depth, *μ*m	2000 (300–7000)
Lymphovascular invasion	30 (20.3)
Positive/unknown vertical endoscopic resection margin	68 (48/20) (45.9)
Positive/unknown lateral endoscopic resection margin	51 (31/20) (34.5)
Reasons for subsequent colectomy	
Poorly differentiated/mucinous histology	7 (4.7)
Positive or unknown vertical margin	68 (45.9)
Positive or unknown lateral margin	51 (34.4)
Lymphovascular invasion	30 (20.3)
Submucosal invasion depth >1000 *μ*m	122 (82.4)
Residual tumor in the colorectal wall on colectomy	6 (4.1)
Lymph node metastasis on colectomy	10 (6.8)

EMR: endoscopic mucosal resection; ESD: endoscopic submucosal dissection. Values are median (range) or number (%).

**Table 2:** Comparison between patients with and those without residual disease (residual tumor in the wall or lymph node metastasis) on colectomy.

Variable	No residual disease (*n* = 132)	Residual disease (*n* = 16)	*p* value
Age, years	60 (35–78)	59 (31–74)	0.889
Men/women	93 (70.5)/39 (29.5)	9 (56.3)/7 (43.7)	0.262
Tumor location			0.530
Cecum and ascending colon	11 (8.3)	0	
Transverse colon	8 (6.1)	1 (6.3)	
Descending colon	4 (3.0)	2 (12.5)	
Sigmoid colon	63 (47.7)	8 (50.0)	
Rectum	46 (34.8)	5 (31.3))	
Size of tumor (histologically measured), mm	13 (4–52)	15 (8–34)	0.682
Macroscopic form of tumor			0.059
Pedunculated	32 (24.2)	0	
Semipedunculated	59 (44.7)	8 (50.0)	
Sessile or flat	41 (31.1)	8 (50.0)	
En bloc resection	108 (81.8)	13 (81.2)	1.0
Differentiation			0.028
Well/moderate	128 (97.0)	13 (81.2)	
Poor/mucinous	4 (1/3) (3.0)	3 (2/1) (18.8)	
Submucosal invasion depth, *μ*m	1800 (300–7000)	2000 (800–4000)	0.342
Lymphovascular invasion	25 (18.9)	5 (31.2)	0.320
Positive/unknown vertical endoscopic resection margin	57 (41/16) (43.2)	11 (7/4) (68.8)	0.047
Positive/unknown lateral endoscopic resection margin	43 (26/17) (32.6)	8 (5/3) (50.0)	0.166

Values are median (range) or number (%).

**Table 3:** Univariate and multivariate analyses of factors associated with residual disease (residual tumor in the wall or lymph node metastasis) on colectomy.

Variables	Univariate OR (95% CI)	*p* value	Multivariate OR (95% CI)	*p* value
Age, years	0.996 (0.945–1.050)	0.888		
Men	0.539 (0.188–1.550)	0.252		
Right-sided colonic location^∗^	0.396 (0.049–3.179)	0.384		
Tumor size	1.013 (0.954–1.075)	0.680		
Sessile type	2.220 (0.779–6.324)	0.136		
Piecemeal resection	0.963 (0.254–3.645)	0.956		
Poor/mucinous histology (versus well/moderate)	7.385 (1.488–36.64)	0.014	7.508 (1.476–38.19)	0.015
Submucosal invasion depth	1.000 (1.000-1.001)	0.342		
Deep submucosal invasion†	1.091 (0.128–9.274)	0.936		
Lymphovascular invasion	0.514 (0.164–1.612)	0.254		
Positive or unknown vertical margin	1.979 (1.005–3.898)	0.048	2.048 (1.003–4.178)	0.049
Positive or unknown lateral margin	1.493 (0.778–2.868)	0.228		

^∗^Right-sided tumor location includes the cecum, ascending colon, and transverse colon. ^†^Deep submucosal invasion means a submucosal invasion depth of >1000 *μ*m. OR: odds ratio; CI: confidence interval.
